# An Unusual Case of Metastatic Gastric Cancer Presenting with Right Heart Failure and Cardiac Metastasis

**DOI:** 10.3390/medicina61020170

**Published:** 2025-01-21

**Authors:** Ebru Engin Delipoyraz, Maral Martin Mildanoglu, Barış Sürül, Oktay Olmuşçelik, Korhan Erkanlı, Ahmet Bilici

**Affiliations:** 1 Department of Medical Oncology, Faculty of Medicine, Istanbul Medipol University, İstanbul 34810, Turkey; maral.mildanoglu@medipol.edu.tr (M.M.M.); abilici@medipol.edu.tr (A.B.); 2 Department of Internal Medicine, Faculty of Medicine, Istanbul Medipol University, İstanbul 34810, Turkey; surulbaris@gmail.com (B.S.); oolmuscelik@medipol.edu.tr (O.O.); 3 Department of Cardiovascular and Thoracic Surgery, Faculty of Medicine, Istanbul Medipol University, İstanbul 34810, Turkey; kerkanli@gmail.com

**Keywords:** cardiac metastasis, gastric cancer

## Abstract

Cardiac metastasis is rarely detected in oncology practice. Herein we present a rare case of metastatic gastric cancer that metastasized to the right atrium and presented with right heart failure. A 51-year-old male patient with no known chronic disease presented with fatigue, abdominal distension and leg edema for 3 weeks. Physical examination revealed abdominal ascites, tachycardia and pretibial edema. Transthoracic echocardiography (TTE) revealed a hypoechoic, less-mobile mass that almost completely filled the right atrium. Moreover, 18F-fluorodeoxyglucose (18F-FDG) positron emission tomography/computed tomography (PET/CT) showed metastatic lesions and a primary tumor-suspicious area in the esophagogastric component. Upper GI endoscopic evaluation performed on the patient revealed an ulcerovegetating mass consistent with gastric adenocarcinoma. A human epidermal growth factor receptor 2 (HER-2) was positiveand programmed death-ligand 1 (PD-L1) combined positive score (CPS) was detected as 15 in immunohistochemistry (IHC). Thereafter, an anticoagulant treatment was started including pembrolizumab and trastuzumab every three weeks, and an oxaliplatin and 5-FU-based chemotherapy regimen was started every two weeks. There was no regression in the cardiac lesion during follow-up; thereafter, there was a significant risk of cardioembolic complications, and a 10 × 7 cm mass filling the right atrium and adhering to the inferior vena cava was resected. The pathology results of the excision material reported gastric carcinoma metastasis. Systemic evaluation performed 3 months later showed regression in primary and metastatic lesions. Cardiac metastases are rare and may not be discovered until autopsy due to the prominence of primary disease findings. Cardiac metastasis, although rare, should be kept in mind in gastric cancer patients presenting with heart failure.

## 1. Introduction

Cardiac metastases, while relatively rare, can originate from a diverse range of primary malignancies and are frequently asymptomatic, with over 90% of cases being clinically silent. Therefore, they are usually not detected during the course of the disease [1]. The frequency of cardiac metastases has been evaluated in postmortem series, with the largest patient groups in different studies reporting frequencies of 2.3% and 9.1% [2,3]. Tumors with a high rate of metastasis to the heart include pleural mesothelioma, malignant melanoma, lung cancer, breast cancer, lymphoproliferative neoplasms, esophageal cancer, and kidney cancer [1,2,4,5]. The rate of metastasis to the heart in gastric carcinoma has been found to be 8% [2].

Extracardiac malignant tumors can spread to the heart in four ways: direct invasion, the hematogenous route, the lymphatic route, and intracavitary spread via the inferior vena cava [4]. Lymphatic spread leads to pericardial metastases, while hematogenous spread primarily leads to myocardial metastases. Endocardial tumor deposits are rare. The clinical symptoms of cardiac metastases are highly variable and vary depending on the site of involvement and tumor burden. Although cardiac metastasis can be the first or even only sign of an undiagnosed malignant tumor, it is usually not detected when the patient is alive and is diagnosed postmortem [6].

Cardiac metastasis can cause a rapid increase in heart size due to pericardial effusion, newly developed heart failure or valvular disease, and atrial or ventricular conduction defects. Findings such as dyspnea, tachypnea and heart murmur, peripheral edema, pleural or pericardial effusion or ascites may occur [7].

Since patients with cardiac metastases usually have disseminated disease, treatment is usually primary tumor treatment or supportive care and palliative care. Although the prognosis of these patients is poor, surgical treatment should be considered when severe obstructive symptoms outweigh the risk of death from surgery and the benefit of medical treatment alone [6]. In this report, we present a case of metastatic gastric cancer presented with heart failure and cardiac metastasis at the initial diagnosis.

## 2. Case Presentation

**Figure 1:**
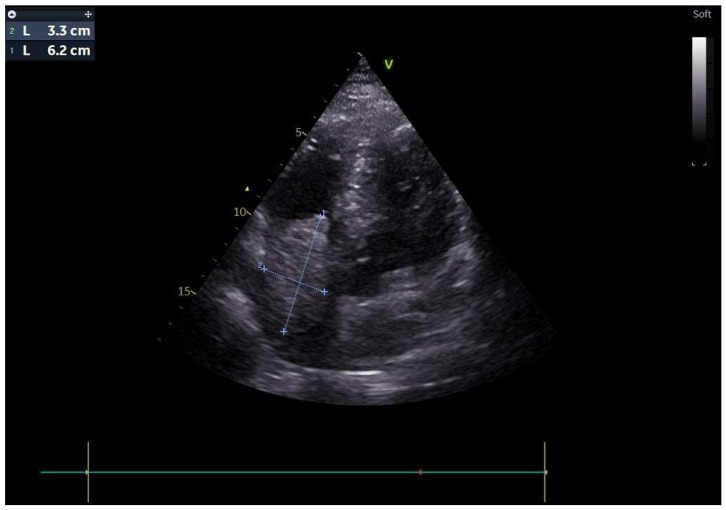
Transthoracic echocardiography (TTE) showed of a low-moving hypoechoic thrombus or mass.

**Figure 2:**
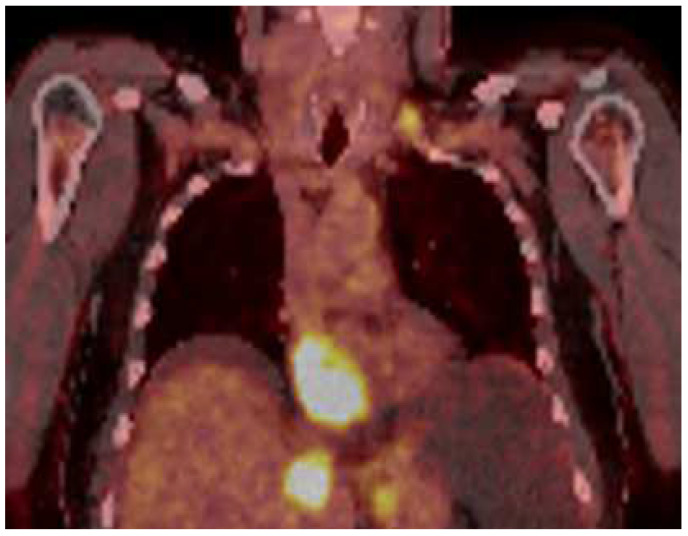
PET scan showed that there was a tumoral lesion with dimensions of 8.8 × 5.5 cm at the level of the right atrium with SUV max: 12.8.

A 51-year-old male patient presented to our clinic in December 2023 with fatigue, abdominal distension and leg edema, which started three weeks prior. He had no history of chronic disease. On physical examination, dullness in the abdomen upon percussion, suggestive of fluid, was observed. Additionally, there were decreased breath sounds in the basals of the lungs, a tachycardic heart rhythm, and significant pretibial edema. The electrocardiogram (ECG) was consistent with sinus tachycardia. The patient’s blood pressure was 130/90 mmHg. Laboratory tests revealed hemoglobin: 13.2 g/dL; fasting blood glucose: 95 mg/dL; serum creatinine 1.2 mg/dL; aspartate aminotransferase (AST) 49 U/L; alanine aminotransferase (ALT): 46 U/L; bilirubin: 0.69 mg/dL; albumin: 3.6 g/dL; carcinoembryonic antigen (CEA): 43 ng/mL; and carbohydrate antigen (CA) 19-9: 361 U/mL. Electrolyte levels were within normal limits. Urinalysis showed proteinuria of 0.4 g/day. Paracentesis revealed a serum-to-ascites albumin gradient greater than 1.1 g/dL. Cytological examination of the fluid revealed benign cytological findings. TTE revealed a lesion 6.2 × 3.3 cm in diameter, suggestive of a low-moving hypoechoic thrombus or mass (Figure 1). This lesion almost completely filled the right atrium and stuck to the tricuspid valve. Dilation of the inferior vena cava and severe insufficiency of the tricuspid valve were also observed. A work-up was started to differentiate heart failure and a possible cardiac mass. Thorax CT showed multiple nodules in the lung and a suspicious mass in the right atrium. A PET scan showed that there was segmental wall thickening at the stomach and esophagogastric junction, measuring 7.5 cm in size and showing 18-FDG uptake with an SUV max of 22.4, which was evaluated in favor of primary malignancy. Multiple metastatic lymphadenopathy in the abdomen, free fluid in the abdominopelvic region, metastatic lesions in the liver and lung and supraclavicular lymphadenopathy were detected (Figure 2). The lesion seen on TTE was evaluated as a tumoral thrombus suspicious lesion with dimensions of 8.8 × 5.5 cm at the level of the right atrium on PET-CT and a maximum standard unit value (SUVmax) of 12.8. Cardiac magnetic resonance imaging (CMRI) was planned to differentiate the tumor/thrombus, but it could not be performed because the patient could not tolerate lying flat. Cardiology and surgery opinions were obtained, and close follow-up with anticoagulant treatment was recommended. An ulcerovegetating mass was detected in the upper-GI endoscopic evaluation. Histopathological evaluation of the biopsy specimen was consistent with gastric adenocarcinoma. PD-L1 IHC was conducted using a Ventana Dako anti-human PD-L1 mouse monoclonal antibody, clone 22C3, with a positive control. PD-L1 protein expression was assessed using the CPS and found to be positive, with a value of 15. HER-2 status was evaluated by both IHC and silver in situ hybridization (SISH). For IHC, Ventana anti-HER-2/neu rabbit monoclonal antibody clone: 4B5 was employed with a positive control. SISH was performed on a Ventana Benchmark Ultra fully automated system using the inform HER-2 dual in situ hybridization DNA probe cocktail with positive and negative controls. The HER-2 IHC score was +3, and SISH demonstrated HER-2 gene amplification with a HER-2/CEP17 ratio ≥ 2 (positive).

**Figure 3:**
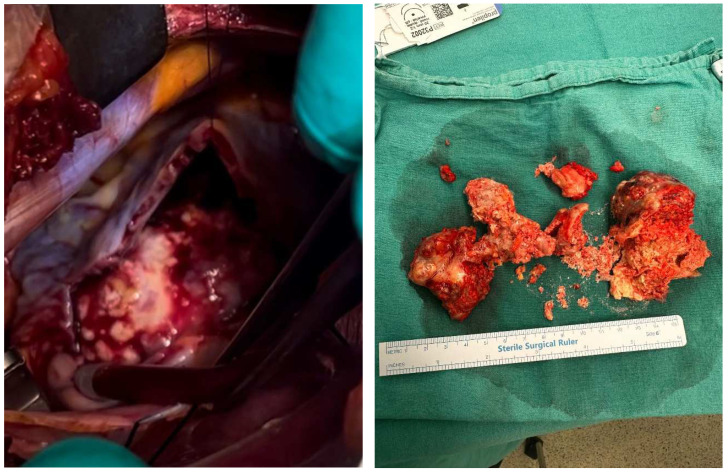
Cardiac mass excision (intraoperative and postoperative).

A combination regimen of pembrolizumab (200 mg IV every 3 weeks), trastuzumab (6 mg/kg IV every 3 weeks after an 8 mg/kg loading dose), and FOLFOX chemotherapy was initiated. The FOLFOX regimen comprised oxaliplatin (85 mg/m^2^), leucovorin (400 mg/m^2^ over 120 min), bolus 5-FU (400 mg/m^2^), and a continuous infusion of 5-FU (2400 mg/m^2^ over 46 h) every 2 weeks. However, trastuzumab could not be given due to cardiac pathology in the first cycle. Twelve cycles of chemotherapy were administered. Treatment with trastuzumab and pembrolizumab continued every 3 weeks until disease progression. Surgical excision was planned due to the lack of regression in the cardiac lesion during follow-up under treatment, the patient’s heart failure clinic and the risk of sudden death. A 10 × 7 cm mass, which completely filled the right atrium and extended to the tricuspid valve, adhering to the inferior vena cava, was resected during a surgical procedure performed in January 2024 (Figure 3).

Histopathological evaluation of the excision material revealed gastric adenocarcinoma metastasis. During the postoperative follow-up, the patient experienced clinical relief and the regression of abdominal ascites and edema. One month after surgery, trastuzumab was added to his treatment. Following six cycles of chemotherapy, an 18F-FDG PET/CT scan demonstrated a partial response, as assessed by Response Evaluation Criteria in Solid Tumors version 1.1 (RECIST v1.1). There was a significant regression in the size and number of pulmonary nodules. The primary lesion and abdominal lymphadenopathy exhibited decreased metabolic activity and reduced size. Hepatic metastases demonstrated near-complete metabolic response. Previously identified free fluid in the abdominopelvic region was no longer observed.

The patient’s progression-free survival (PFS), defined as the time from initiation of first-line systemic therapy to disease progression, was 10 months. The patient remains under follow-up, and overall survival (OS), defined as the time from initiation of systemic therapy to death, has not yet been reached. The patient is continuing to receive treatment and follow-up.

## 3. Discussion

Cardiac metastases usually occur in the late period in solid tumors and are rarely the first site of metastasis to present symptoms [8]. In our case, the patient, who had no previous known malignancy, presented with findings of right heart failure due to intracavitary cardiac metastasis of gastric adenocarcinoma.

Cardiac metastases most commonly involve the pericardium (64–69%), followed by the epicardium (25–34%) and the myocardium [9]. Cardiac tumors can manifest with a wide range of symptoms, including hemodynamic compromise, arrhythmias, pericardial effusions, and systemic embolization. Heart failure and thromboembolism are the most common presentations, often requiring urgent surgical intervention [10]. Cardiac metastasis can cause a rapid increase in heart size due to pericardial effusion, newly developed heart failure or valvular disease, and atrial or ventricular conduction defects. Findings such as dyspnea, tachypnea and heart murmur, peripheral edema, pleural or pericardial effusion or ascites may occur [7]. Although ECG evaluation is valuable, it often shows nonspecific ST-T changes [11]. In our case, the patient had bilateral 3+ pretibial edema and widespread free fluid in the abdomen. The patient’s symptoms were interpreted as being due to right ventricular failure caused by an obstruction of the right ventricular inflow tract by a metastatic deposit from the gastric adenocarcinoma, as evidenced by biochemical and imaging studies.

Tumors metastasize to the heart in four different ways. While locally aggressive mediastinal and pleural tumors invade the pericardium directly, breast and lung cancer often involve the pericardium and epicardium via the lymphatic route. While malignant melanoma, lymphoma and sarcomas usually cause myocardial and endocardial metastasis via a hematogenous route, tumors such as renal cell carcinoma and hepatocellular carcinoma can extend to the inferior vena cava and spread to the right atrium via a transvenous route [12,13]. Endocardial and intracavitary metastases are rare and account for 3% to 5% of cardiac metastases at autopsy [2,3]. However, such intracavitary metastases may lead to important clinical consequences. As in our case, right heart failure may occur when right atrium metastases block the right ventricular inlet. Additionally, cardioembolic complications due to tumor embolism can occur in the form of stroke due to left-sided heart metastases or pulmonary embolism due to right-sided heart metastases [12].

While primary cardiac tumors are rare, cardiac metastases, especially from extracardiac primaries, are more common. Despite this, surgical resection is infrequently performed. Imaging modalities such as CT and MRI are valuable for assessing the anatomy of intrapericardial tumors and their invasion of cardiac structures, while echocardiography is the gold standard for intracavitary and mural cardiac tumors [10]. TTE is the first imaging method in the diagnosis of cardiac metastases. For many tumors, TTE provides information about the location, size, mobility, and hemodynamic status of the cardiac mass. A sensitivity of over 90% can be achieved in differentiating benign from malignant processes using 18F- FDG PET/CT [14]. Recent advances in CT and CMRI may aid in more detailed characterizations of the cardiac mass [15]. In our case, 18F-FDG PET/CT and TTE were used for cardiac imaging and detailed definitions of the metastatic mass. With the current findings, additional imaging or cardiac biopsy was not needed in the patient whose primary tumor was known to be metastatic. TTE monitoring was performed at short intervals from cardiac mass detection to surgery. This method may have been clinically beneficial in making surgical decisions in terms of the practicality of application and the guiding effectiveness of follow-up with the same method. However, during this period, the risk of tumor embolism and sudden death and the patient’s right heart failure symptoms made the treatment decision challenging.

Cardiac metastases are usually seen in cancer patients with multiple metastases and extensive disease burden. Therefore, the most important goals of intervention are symptom relief and the prevention or delay of symptom recurrence. Surgical resection is generally preferred for cases where the prognosis is good, cases where the tumor can be completely removed, or specifically cases of intracardiac obstruction [12]. Our patient was initially managed conservatively with close follow-up. However, due to the refractory nature of the patient’s heart failure symptoms, surgical intervention was pursued. Post-operatively, the patient exhibited a marked improvement in symptoms, enabling the continuation of optimal medical management. The clinical course of our patient is consistent with the reported outcomes in the literature [13].

Since gastric cancer is usually diagnosed at an advanced stage, systemic therapy is the mainstay of treatment in these patients [16]. Our patient was a case of advanced gastric adenocarcinoma with widespread metastases, and systemic treatment, chemotherapy, immunotherapy, and targeted therapies were the primary goals of treatment. Studies have shown that OS increased from 11.1 months to 13.8 months by adding trastuzumab to chemotherapy in HER-2-positive patients [17] and that median OS increased to 20 months by adding pembrolizumab to chemotherapy and trastuzumab, especially in patients whose tumors had a PD-L1 CPS of 1 or higher [18]. Because our patient’s intracardiac metastasis will determine OS independently of the spread of the gastric cancer due to the risk of obstruction and sudden death, we think that the surgery contributed to our patient’s prognosis.

## 4. Conclusions

Cardiac metastases are clinically silent and warrant evaluation for cardiac involvement, especially in patients with known malignancy and cardiac symptoms. Rarely, gastric cancer may present with symptoms related to cardiac metastasis, like our patient. Although the treatment of the primary disease and palliative care are at the forefront for secondary cardiac tumors, surgical intervention may be considered in cases with high cardiac risk. This decision should be made after carefully evaluating the potential benefit of surgery on the patient’s prognosis. A multidisciplinary approach is beneficial in these patients. This approach ensures optimal care by involving specialists from various fields. Cardiac metastasis, although rare, should be kept in mind in gastric cancer patients presenting with the symptoms of heart failure.

## Data Availability

Data is contained within the article.
